# Impact of vaccination against severe COVID-19 in the French population aged 50 years and above: a retrospective population-based study

**DOI:** 10.1186/s12916-023-03119-8

**Published:** 2023-11-08

**Authors:** Laetitia Tan-Lhernould, Cynthia Tamandjou, Guilhem Deschamps, Johnny Platon, Cécile Sommen, Fanny Chereau, Isabelle Parent du Châtelet, Simon Cauchemez, Sophie Vaux, Juliette Paireau

**Affiliations:** 1 Direction des Maladies Infectieuses, Santé publique France, 12 Rue du Val d’Osne, Saint-Maurice, 94415 France; 2 Direction Appui, Traitements et Analyses de données, Santé publique France, 12 Rue du Val d’Osne, Saint-Maurice, 94415 France; 3 Mathematical Modelling of Infectious Diseases Unit, Institut Pasteur, Université Paris Cité, CNRS UMR 2000, Paris, F-75015 France

**Keywords:** COVID-19, Vaccination, Impact, Mortality, Hospitalization, Burden

## Abstract

**Background:**

Given the widespread implementation of COVID-19 vaccination to mitigate the pandemic from the end of 2020, it is important to retrospectively evaluate its impact, in particular by quantifying the number of severe outcomes prevented through vaccination.

**Methods:**

We estimated the number of hospitalizations, intensive care unit (ICU) admissions and deaths directly averted by vaccination in France, in people aged ≥ 50 years, from December 2020 to March 2022, based on (1) the number of observed events, (2) vaccination coverage, and (3) vaccine effectiveness. We accounted for the effect of primary vaccination and the first booster dose, the circulating variants, the age groups, and the waning of vaccine-induced protection over time.

**Results:**

An estimated 480,150 (95% CI: 260,072–582,516) hospitalizations, 132,156 (50,409–157,767) ICU admissions and 125,376 (53,792–152,037) deaths were directly averted by vaccination in people aged ≥ 50 years, which corresponds to a reduction of 63.2% (48.2–67.6), 68.7% (45.6–72.4) and 62.7% (41.9–67.1) respectively, compared to what would have been expected without vaccination over the study period. An estimated 5852 (2285–6853) deaths were directly averted among the 50–59 years old, 16,837 (6568–19,473) among the 60–69 years old, 32,136 (13,651–36,758) among the 70–79 years old and 70,551 (31,288–88,953) among the ≥ 80 years old.

**Conclusions:**

The vaccination campaign in France considerably reduced COVID-19 morbidity and mortality, as well as stress on the healthcare system.

**Supplementary Information:**

The online version contains supplementary material available at 10.1186/s12916-023-03119-8.

## Background

Following its emergence in Wuhan, China, in December 2019, the coronavirus disease 2019 (COVID-19), caused by severe acute respiratory syndrome coronavirus 2 (SARS-CoV-2), quickly spread around the globe. In France, the pandemic has caused more than 160,000 deaths in hospitals and nursing homes and heavily impacted the healthcare system, with more than one million hospitalizations and more than 150,000 admissions in intensive care units (ICU), as of 23 March 2023. During the first year of the pandemic, non-pharmaceutical interventions such as lockdowns and curfews were the primary means to control the spread of the disease [1]. These interventions proved effective at reducing transmission [1–4] but came with considerable economic costs and broader impact on society. The quick development of highly effective vaccines against severe forms of disease [5], available by the end of 2020, has brought an alternative means to mitigate the pandemic.

In France, four different vaccines were initially used, two mRNA vaccines (Pfizer/BioNTech BNT162b2 and Moderna mRNA-1273) and two viral vector vaccines (Oxford/AstraZeneca ChAdOx1-S and Johnson & Johnson Ad26.COV2.S). The two mRNA vaccines were the most widely used in France and required a two-dose schedule for primary vaccination. The French vaccination campaign started in December 2020, first targeting high-risk populations such as the elderly people in nursing homes and healthcare workers. It was then progressively extended to larger groups of the population, to finally target the whole adult population in May 2021. Due to waning of vaccine-induced immunity, a first booster dose was recommended from September 2021 onwards, followed by a second booster in March 2022 for high-risk groups. As of 23 March 2023, more than 90% of the adult population has received a full primary vaccination, and 75% has received the first booster dose.

Given the widespread deployment of COVID-19 vaccination, it is important to retrospectively evaluate its impact, in particular by quantifying the number of severe outcomes prevented through mass vaccination. A few studies have estimated the impact of COVID-19 vaccination in terms of averted morbidity and mortality [5–9]. One study provided estimates specific to France [6]. This study covered the period December 2020–November 2021, focusing on the number of deaths averted by the primary series (no booster) in people aged 60 years and above, and it assumed that vaccine effectiveness (VE) was the same for Alpha and Delta variants, for all age groups, and that there was no waning of protection over time.

The objective of our study was to estimate the number of hospitalizations, ICU admissions and deaths averted by vaccination in France between December 2020 and March 2022, during the most acute phase of the pandemic when there was concern that circulation of SARS-CoV-2 in France might saturate hospitals. The study covered the periods of circulation of Alpha, Delta and Omicron BA.1 variants. Although Omicron BA.1 was reported to be less severe than Delta [10], it was responsible for a large epidemic wave in France between December 2021 and March 2022, due to an increased transmissibility [11]. This was also the period during which the first booster dose was introduced in France. It was thus important to quantify the impact of vaccination on severe outcomes during the Omicron period. We focused on the population aged 50 years and above, which was the most affected by severe COVID-19 in France: this population accounted for 80% of hospitalizations and 99% of deaths. As vaccine effectiveness can vary with age and variant [12], we stratified the analysis in order to account for different values of VE according to age groups and variants. In addition, we improved the methodology usually applied for this type of study by accounting for the waning of vaccine protection over time. This new methodological development could be useful in various settings (e.g. other countries or diseases), in particular when the mean delay since vaccination is long or the decline of vaccine protection is fast.

## Methods

### Study design

We conducted a retrospective ecological study based on French national surveillance data (see Data sources), from December 28, 2020 (week 53–2020 — start of the vaccination campaign) to March 6, 2022 (week 9–2022 — end of Omicron BA.1 wave). The study population was the French population aged 50 years and above, stratified into four age groups: 50–59, 60–69, 70–79 and 80 years old and above. We estimated the impact of the first and second doses of primary vaccination and the first booster, in terms of the number of averted hospitalizations (admissions to all types of hospitalization services), ICU admissions and deaths.

### Methods

To compute the number of averted events, we used a method initially developed for tuberculosis [13], which was later applied to influenza [14, 15] and more recently to COVID-19 [6, 7]. Details are given in Additional file 1: Text S1. Basically, the number of averted events (${N}_{\mathrm{averted}}$) can be estimated from the number of observed events (${N}_{\mathrm{observed}}$), vaccine coverage ($VC$) and vaccine effectiveness ($VE$), as follows:

1 $${N}_{\mathrm{averted}}= {N}_{\mathrm{observed}} \times \frac{VC\times VE}{1 - (VC \times VE)}$$

where the term $VC\times VE$ represents the proportion of the population that is protected by vaccination. This method only accounts for the direct effects of vaccination on severe outcomes (not its indirect effects such as impact on transmission) and therefore provides a lower bound estimate of the true number of averted events. Besides, in a scenario without vaccination, it is probable that additional control measures would have been implemented, which we do not account for here (i.e. we estimate the impact of vaccination under the assumption that the same NPIs would have been implemented over the study period). These two points will be further addressed in the discussion.

This formula can be extended to account for the week of observation ($w$) and the number of doses received ($k$, ranging from one to three (two doses and one booster)) [6, 15]:

2 $${{N}_{\mathrm{averted}}}_{w}= {{N}_{\mathrm{observed}}}_{w} \times \frac{\sum_{k=1}^{3}{VC}_{w,k} \times {VE}_{k}}{1 - (\sum_{k=1}^{3}{VC}_{w,k} \times {VE}_{k})}$$

where ${VC}_{k}$ represents the vaccine coverage of *exactly* $k$ doses (not *at least* $k$ doses).

This formula assumes that VE is the same for all vaccinated individuals, regardless of the time elapsed since vaccination. However, VE is not constant over time: it quickly increases in the first weeks following vaccination due to the build-up of immunity and declines over time due to waning immunity [16–19]. In order to account for the evolution of VE according to the time elapsed since vaccination $\left(\Delta \right)$ in weeks, we modified the formula as follows:

3 $${{N}_{\mathrm{averted}}}_{w}= {{N}_{\mathrm{observed}}}_{w} \times \frac{\sum_{k=1}^{3}\sum_{\Delta =0}^{w-1}{VP}_{w,k,\Delta } \times {VE}_{k, \Delta }}{1 - (\sum_{k=1}^{3}\sum_{\Delta =0}^{w-1}{{VP}_{w,k, \Delta } \times VE}_{k, \Delta } )}$$

where ${VE}_{k, \Delta }$ is the vaccine effectiveness of the $k$^th^ dose $\Delta$ weeks after vaccination, and ${VP}_{w,k,\Delta }$ represents the proportion of people who received their last dose $k$ exactly $\Delta$ weeks before the week of observation $w$. Of note, the sum of ${VP}_{w,k,\Delta }$ over all $\Delta$ corresponds to the vaccine coverage of exactly $k$ doses on week $w$ ($\sum_{\Delta =0}^{w-1}{VP}_{w,k,\Delta }={VC}_{w,k})$.

This formula was applied separately for each age group and each variant, in order to account for different VE according to age groups and variants. The total number of events directly averted by vaccination (number of hospitalizations, ICU admissions and deaths) was then obtained by summing the number of averted events over all weeks, age groups and variants. All analyses were conducted in R software version 4.1.2 (R Foundation, Vienna, Austria).

### Data sources

For the number of observed events, we relied on hospitalization and death data from the SI-VIC database, maintained by the ANS (Agence du Numérique en Santé) and sent daily to Santé publique France, the French national public health agency. This database provides real-time data on patients hospitalized for COVID-19 in French public and private hospitals, including their age, date of hospitalization, type of hospitalization services and outcome (discharged/deceased). All COVID-19 cases are either biologically confirmed or present with a computed tomographic image highly suggestive of SARS-CoV-2 infection. For hospitalizations, we included patients hospitalized in all types of services (general wards, ICU, long-term care and rehabilitation, emergency care…) and excluded patients hospitalized for reasons not linked to a COVID-19 infection. For deaths, we included all patients deceased in the hospitals with a COVID-19 infection and added individuals deceased in nursing homes (resident homes for elderly) with a COVID-19 infection from the SurvESMS database. The SurvESMS database, administered by Santé publique France, was designed for the monitoring of COVID-19 cases and deaths among the residents of nursing homes; it allows the distinction between residents who died in nursing homes from those who died in hospitals (already accounted for in the SI-VIC database). In the absence of data on age, individuals were considered 80 + years old; this assumption was based on 2019 data which showed that the mean age of people deceased in nursing homes was 89 years old [20]. Deaths at home could not be accounted for. The time series of hospitalizations, ICU admissions and deaths according to each variant were reconstructed using the SI-DEP database, the national surveillance system describing SARS-CoV-2 RT-PCR and antigen test results arising from all private and public French laboratories (see details in Additional file 1: Text S2). The three databases (SI-VIC, SurvESMS and SI-DEP) are intended to be exhaustive.

Regarding vaccine coverage, we used the VAC-SI database, the national information system developed by the French Health Insurance to monitor the implementation of COVID-19 vaccination campaigns, since the start of vaccine distribution in December 2020, across the country. Individual data include the number of doses, the date of vaccination, the type of vaccine, and socio-demographic information such as age. All types of vaccine were considered (Pfizer/BioNTech BNT162b2, Moderna mRNA-1273, Oxford/AstraZeneca ChAdOx1-S and Johnson & Johnson Ad26.COV2.S). For each week of observation $w$ and each age group, the number of vaccinated individuals according to the number of doses received before the week of observation $w$ and to the week of vaccination (in order to compute the time elapsed since vaccination, $\Delta$) were extracted. For each individual, on a given week of observation $w$, only the last dose received before that week was taken into account, to avoid counting the same person twice: e.g. when an individual received a second dose on week $w$, they were no longer counted among the first-dose individuals from week $w$ onwards (they were only counted among the first-dose individuals up to week $w-1$). We excluded individuals for which the date of the first dose was posterior to the date of the second dose or the booster dose, or the date of the second dose was posterior to the date of the booster. For the denominator (number of individuals per age group in the French population), we used 2022 demographic data from the National Institute of Statistics and Economic Studies (INSEE).

With regards to the effectiveness of COVID-19 vaccines against COVID-19 variants, we extracted data from a recent VE study conducted by Santé publique France [12]. The study investigated VE of the two mRNA vaccines Pfizer/BioNTech BNT162b2 and Moderna mRNA-1273 against Alpha (unpublished data), Delta and Omicron BA.1 severe outcomes (general ward hospitalizations and ICU/deaths) among immunocompetents ≥ 50 years old French individuals, between January 11, 2021, and February 10, 2022. Analyses were performed according to 50–79 and 80 years old and above age groups, and the number of vaccine doses (one dose, two doses and the first booster dose). VE according to time since vaccination were also estimated. These estimates were smoothed to remove random fluctuations over time.

Note that VE against Omicron severe outcomes were sourced from multiple studies available in the literature: the aforementioned French study for VE of the first dose against ICU/deaths [12], a test-negative case–control study in England for VE against hospitalizations among people aged 65 years and older (which we applied to our 50 + population) [21], and a test-negative case–control study in Canada for VE of the second dose and booster against deaths among people aged 18 years and older (which we applied to our 50 + population) [16].

In these three studies, the date associated to an individual is the date of the test or the date of symptom onset. In order to account for the time between the test or symptom onset and the event of interest (hospitalization, ICU admission or death), we applied a lag of 1 week to VE against hospitalization and ICU admission and a lag of two weeks to VE against death. Finally, a linear decay of VE was assumed to account for the waning of immunity at longer time horizons. The decay values were extracted from the literature and set to − 0.5 points/week for the second dose after 21 weeks [22, 23] and − 0.4 points/week for the booster dose after 21 weeks [24]. In the absence of data from the literature for the first dose, we applied a coefficient twice higher than for the second dose (− 1 point/week). This coefficient was applied from the 13th week after vaccination [25]. In order to estimate uncertainty around the number of averted events, we used the 95% confidence intervals of VE estimates published in the three aforementioned studies [12, 16, 21].

## Results

### Data description

#### Number of observed events

**Fig. 1:**
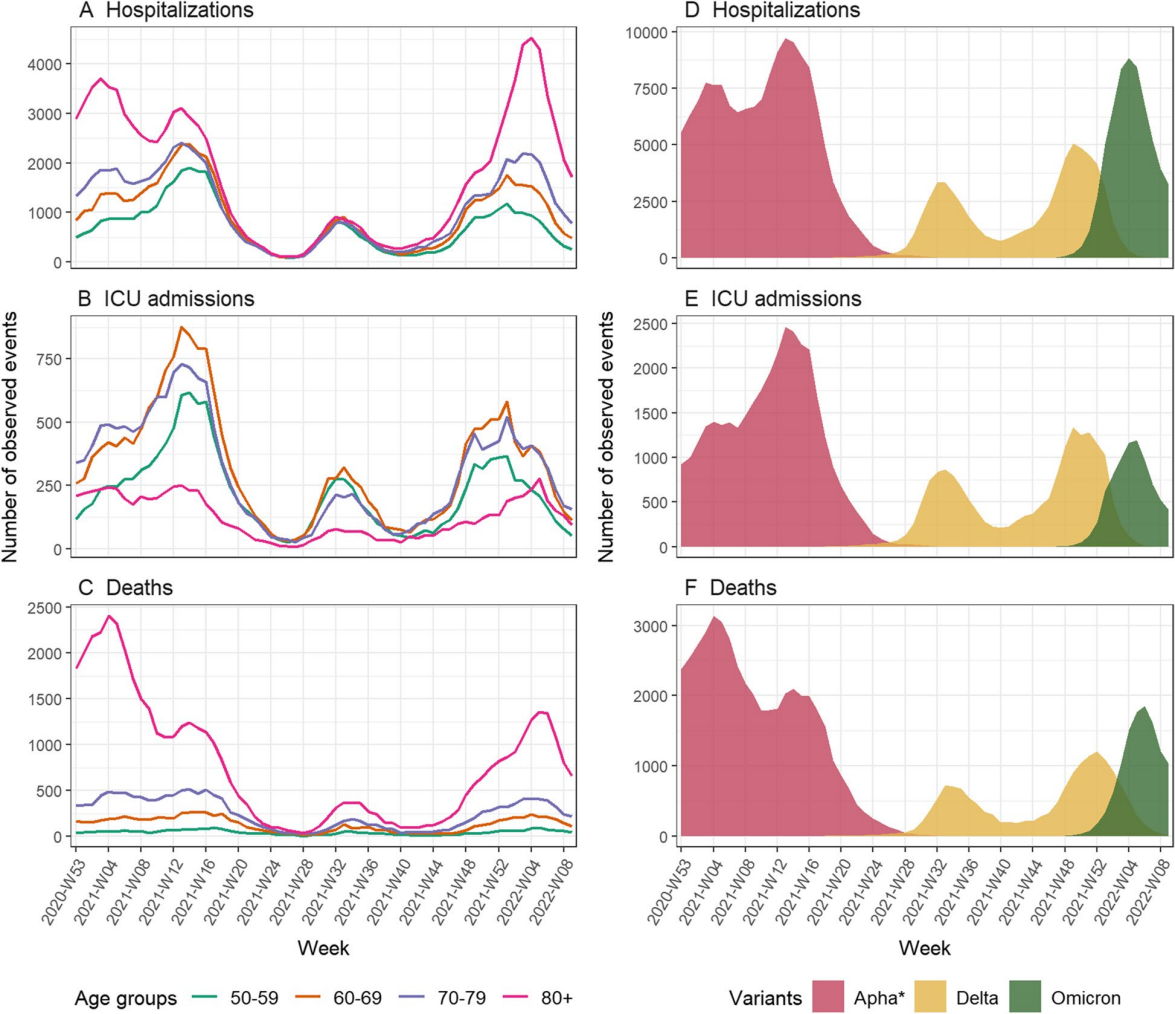
Number of observed COVID-19 events by age and variant: **A** hospitalizations by age, **B** intensive care unit (ICU) admissions by age, **C** deaths by age, **D** reconstructed hospitalizations by variant, **E** reconstructed ICU admissions by variant, **F** reconstructed deaths by variant. *At the beginning of the time period, for simplification purposes, all cases were attributed to the Alpha variant although the historical strain was still circulating (see Additional file 1: Text S2)

Over the study period (December 28, 2020, to March 6, 2022), 279,443 hospitalizations, 60,186 admissions in ICU and 74,690 deaths were observed in France among the population aged 50 years and above. During the first five months of the study period, the Alpha variant was the main circulating variant, reaching a peak of 9731 weekly hospitalizations in March 2021 (Fig. 1). It was then replaced by the Delta variant, with a first peak of 3349 weekly hospitalizations in August 2021 and a second peak of 5074 weekly hospitalizations in December 2021. Omicron BA.1 emerged in November 2021 and quickly replaced Delta to reach a peak of 8845 weekly hospitalizations in January 2022. People aged 80 years and older represented 38.8% (108,399) of hospitalizations, 12.8% (7,677) of ICU admissions and 64.4% (48,088) of deaths (Fig. 1). The most represented age group in ICU admissions was the 60–69 years old, with 20,175 (33.5%) ICU admissions.

#### Vaccine coverage

In total, 25,152,788 people aged 50 years and above received at least one vaccine dose against COVID-19 over the study period and met our inclusion criteria (Additional file 1: Fig. S1). Vaccine coverage of at least one dose reached 10% in week 8–2021 and 92.1% at the end of the study period (week 9–2022) (Additional file 1: Fig. S2). Vaccine coverage of at least two doses reached 10% in week 12–2021 and 89.5% at the end of the study period. After the vaccination campaign for the first booster was launched in week 35–2021, vaccine coverage for this booster dose progressively increased and reached 79.2% at the end of the study period.

#### Vaccine effectiveness

**Fig. 2:**
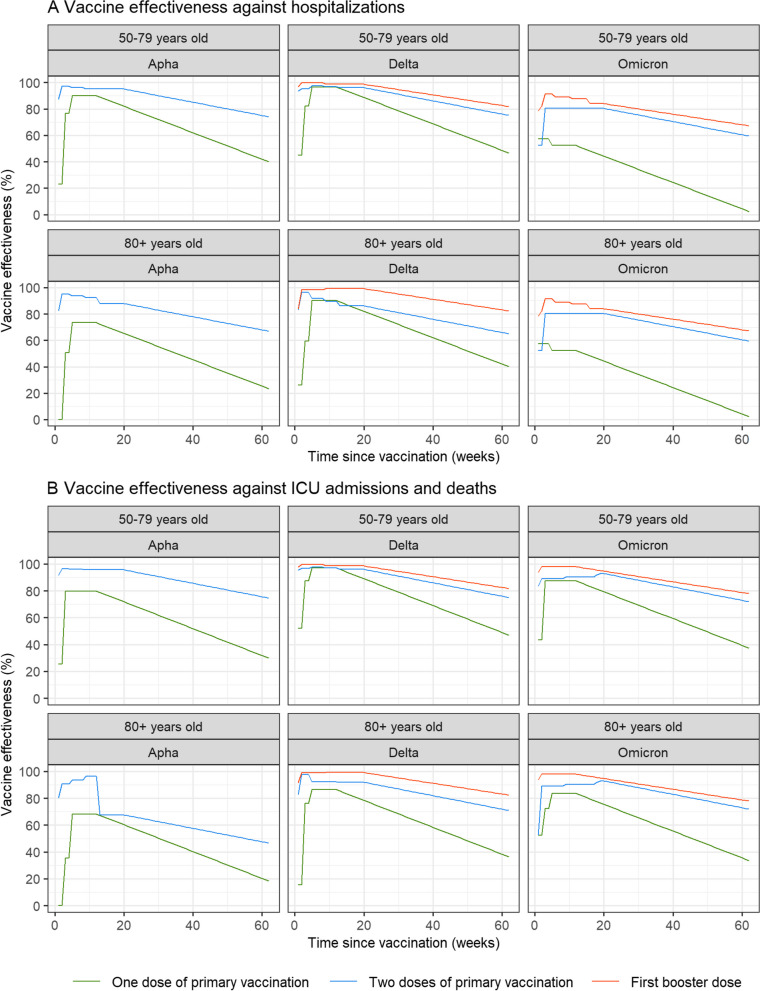
Values of vaccine effectiveness against hospitalizations (**A**) and ICU admissions and deaths (**B**), by dose, age and variant, according to the time since vaccination, that were used in our study. Estimations extracted from sources described in the “Methods” section (Tables S1, S2 and S3) were combined, smoothed and extended over time based on a linear decay (Methods)

Figure 2 presents the values of VE that were used in our study (based on estimations extracted from sources described in Methods and presented in Additional file 1: Tables S1, S2 and S3). Overall, VE quickly increased in the first weeks following vaccination, reached a plateau and then decreased over time. In general, it was higher (1) for a higher number of doses, (2) for 50–79 years old compared to 80 years old and above (when data by age was available), (3) for Alpha and Delta compared to Omicron, and (4) for ICU admissions and deaths compared to hospitalizations. For instance, regarding protection against ICU admissions and deaths, the maximum VE of one dose for the Alpha variant was 80% for 50–79 years old vs 68% for 80 years old and older (5–12 weeks after vaccination), and decreased to 30% vs 20% 62 weeks after vaccination. For Delta, VE against ICU admissions and deaths among the 50–79 years old was high for all doses (maximum > 98%) and decreased to 47%, 75% and 82% 62 weeks after vaccination with the first dose, the second dose and the first booster, respectively. With respect to Omicron, VE of the booster dose against hospitalizations peaked at 91%, followed by a decline to 67% 62 weeks after vaccination, while VE of the booster dose against ICU admissions and deaths was 98% 2 to 12 weeks after vaccination and decreased to 78% 62 weeks after vaccination.

### Estimated number of directly averted events

Overall, we estimated that 480,150 (95% CI: 260,072–582,516) hospitalizations, 132,156 (50,409–157,767) ICU admissions and 125,376 (53,792–152,037) deaths have been directly averted by vaccination in people aged ≥ 50 years, which corresponds to a reduction of 63.2% (48.2–67.6), 68.7% (45.6–72.4) and 62.7% (41.9–67.1) respectively, compared to what would have been expected without vaccination (sum of observed and averted events).

**Fig. 3:**
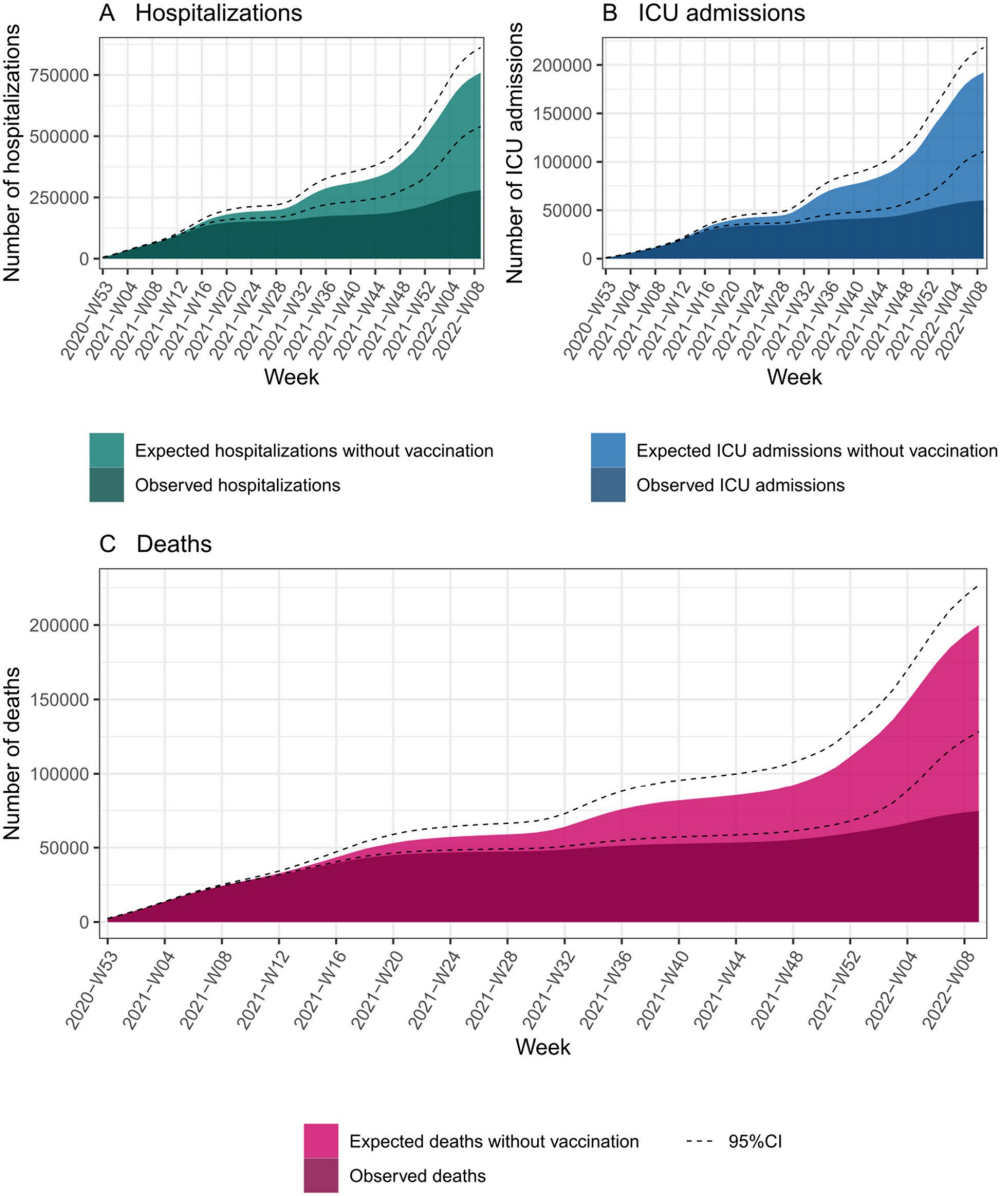
Cumulative numbers of **A** hospitalizations, **B** ICU admissions and **C** deaths observed and expected without vaccination, in the French population aged 50 years and above, from week 53–2020 to week 9–2022. Dashed lines show the 95% confidence intervals of the cumulative numbers of expected events. Non-cumulative curves are shown in Additional file 1: Fig. S3

The impact of the vaccine campaign was already substantial by the end of April 2021 with 4906 averted deaths, and increased along with increased vaccine coverage (Fig. 3). The highest number of averted events was estimated in December 2021–March 2022 (Delta/Omicron wave), due to the high number of observed events combined with the high vaccine coverage for the first booster dose during this time period. The estimated percentage of averted events was 21.7%, 20.4% and 19.1% for hospitalizations, ICU admissions and deaths respectively during the circulation of the Alpha variant. This percentage increased to 79.7%, 81.2% and 78.3% for Delta and 74.7%, 85.4%, and 83.2% for Omicron (Additional file 1: Tables S4, S5 and S6).

When stratifying the analysis by age, we estimated that the percentage of hospitalizations averted by vaccination increased from 62.0% in 50–59 years old to 66.5% in 70–79 years old and then decreased to 60.5% in 80 years old and older (Table 1 and Additional file 1: Table S4). A similar trend was observed for ICU admissions (Table 1 and Additional file 1: Table S5). For deaths, the reduction was similar in all age groups (67.3%), but in 80 years old and older among whom the percentage of deaths averted due to vaccination decreased to 59.5% (Table 1 and Additional file 1: Table S6). We estimated that 5852 (2285–6853) deaths have been averted among the 50–59 years old, 16,837 (6568–19,473) among the 60–69 years old, 32,136 (13,651–36,758) among the 70–79 years old and 70,551 (31,288–88,953) among the 80 years old and above.

**Table 1:** Estimated number of directly averted events (hospitalizations, ICU admissions and deaths) by age, in the French population aged 50 years and above, from week 53–2020 to week 9–2022

Event	Age group (years)	Number of events		Rate per 100,000		Percentage averted by vaccination (95% CI)
		**Observed**	**Averted by vaccination (95% CI)**	**Observed**	**Expected without vaccination (95% CI)**	
**Hospitalizations**	50–59	43,757	71,526 (37,934–85,122)	492.2	1296.7 (918.8–1449.6)	62.0 (46.4–66.0)
	60–69	59,246	107,642 (57,120–126,159)	734.5	2069.0 (1442.7–2298.6)	64.5 (49.1–68.0)
	70–79	68,041	134,862 (71,915–155,691)	1092.4	3257.5 (2246.9–3591.9)	66.5 (51.4–69.6)
	80 +	108,399	166,120 (93,103–215,544)	2625.4	6648.8 (4880.3–7845.8)	60.5 (46.2–66.5)
	**All (50 +)**	**279,443**	**480,150 (260,072–582,516)**	**1023.1**	**2780.9 (1975.2–3155.7)**	**63.2 (48.2–67.6)**
**ICU admissions**	50–59	13,651	28,074 (9636–33,776)	153.5	469.3 (261.9–533.4)	67.3 (41.4–71.2)
	60–69	20,175	44,790 (16,700–52,992)	250.1	805.4 (457.2–907.1)	68.9 (45.3–72.4)
	70–79	18,683	44,007 (17,595–51,144)	299.9	1006.5 (582.4–1121.0)	70.2 (48.5–73.2)
	80 +	7677	15,285 (6478–19,855)	185.9	556.1 (342.8–666.8)	66.6 (45.8–72.1)
	**All (50 +)**	**60,186**	**132,156 (50,409–157,767)**	**220.3**	**704.2 (404.9–797.9)**	**68.7 (45.6–72.4)**
**Deaths**	50–59	2839	5852 (2285–6853)	31,9	97.8 (57.6–109.0)	67.3 (44.6–70.7)
	60–69	8170	16,837 (6568–19,473)	101,3	310.0 (182.7–342.7)	67.3 (44.6–70.4)
	70–79	15,593	32,136 (13,651–36,758)	250,3	766.3 (469.5–840.5)	67.3 (46.7–70.2)
	80 +	48,088	70,551 (31,288–88,953)	1164.7	2873.4 (1922.5–3319.1)	59.5 (39.4–64.9)
	**All (50 +)**	**74,690**	**125,376 (53,792–152,037)**	**273.4**	**732.5 (470.4–830.1)**	**62.7 (41.9–67.1)**

## Discussion

In this study, we estimated the number of COVID-19 hospitalizations, ICU admissions and deaths directly averted by vaccination between December 2020 and March 2022 among the French population aged 50 years and above. We accounted for different levels of VE as a function of age, variant, vaccine status and type of events, and improved an existing methodology in order to account for the waning of protection over time. Overall, we estimated that vaccination had a strong impact on severe outcomes, preventing 480,150 (260,072–582,516) hospitalizations, 132,156 (50,409–157,767) ICU admissions and 125,376 (53,792–152,037) deaths, i.e. 63.2% (48.2–67.6) of hospitalizations expected without vaccination, 68.7% (45.6–72.4) of expected ICU admissions and 62.7% (41.9–67.1) of expected deaths.

We found that the number of averted hospitalizations and deaths increased with age, following the trend in the number of observed hospitalizations and deaths, due to the increased severity of COVID-19 with age [2, 26, 27]. The 80 + population accounted for 35% of averted hospitalizations and 56% of averted deaths. On the contrary, the number of averted ICU admissions was lower in the 80 + population, because access to critical care was more limited for this population during the pandemic. Overall, 67% of averted ICU admissions were among the 60–79 years old. The percentages of averted events were relatively comparable for the 50–59, 60–69 and 70–79 populations, but slightly lower in the 80 + population, which can be explained by a lower vaccine coverage and a slightly lower VE for the Alpha and Delta variant in this population. Regarding results by variant, we estimated that the percentage of averted events was higher during the circulation of Delta and Omicron (75–85%) compared to Alpha (around 20%), which can be related to higher vaccine coverage during the Delta and Omicron periods compared to the Alpha period. Among the 125,376 averted deaths, we estimated that 58,450 (47%) were averted during the circulation of Delta, and 55,788 (44%) during the circulation of Omicron.

Our findings are in agreement with the study by Meslé et al., which estimated the number of averted deaths in 33 European countries including France, from December 2020 to November 2021, in the population aged 60 years and above [6]. The authors found that 38,715 deaths were averted in France, which is comparable to our estimate of 34,817 averted deaths for the same time period and the same population. The small difference can be explained by differences in assumed VE, which, in our study, were stratified by age and variant, and accounted for the waning of protection over time. The fact that the difference was small suggests that the impact of waning was limited over this time period, probably because the decay rate of mNRA vaccine efficacy against severe outcomes was quite slow and, due to the vaccine schedule, only a few people had very long delays since last dose (see for instance Additional file 1: Fig. S2). With a similar methodology, Sacco et al. found a 39% reduction in expected deaths, in the Italian population aged 60 years and above, from January to September 2021 [7]. This is close to our estimates of 35% averted deaths in France, for the same age group and time period, knowing that the vaccine coverage of primary vaccination was a bit lower in France (80%) compared to Italy (above 85%) by the end of September 2021. In other settings, a US modelling study estimated that 56% of hospitalizations and 58% of deaths were averted in the US population over 18 years between December 2020 and September 2021, with a vaccine coverage at 67% in this population by the end of the study period [8]. In Israel, a study comparing the rates of hospitalizations and deaths between vaccinated and unvaccinated individuals aged 16 years and older, found that two thirds of hospitalizations and deaths were averted in the first four months of the vaccine campaign, with a full vaccination coverage reaching 74% at the end of the study period [5]. A global modelling study estimated a similar reduction in hospitalizations and deaths worldwide (63%) between December 2020 and December 2021 [9]. Compared to our study, this study considered a much lower global vaccine coverage (56% for one dose, 46% for two doses and 4% for the booster), but accounted for both the direct and indirect effects of vaccination while we only considered the direct effects here. Thus, despite differences in the methodological approaches, the studied populations and the levels of vaccine coverage, our findings are in line with previous studies, showing a large impact of vaccination on severe SARS-CoV-2-related outcomes, and extend prior research by providing estimates for the period when Omicron was circulating.

Our study has some limitations, primarily related to the availability and quality of input data and the methodology used. First, our analysis was based on the number of deaths reported by hospitals and nursing homes and therefore does not include deaths that occurred at home. However, as the proportion of deaths that occurred at home is expected to be small, the underestimation of the number of observed and avoided deaths should be minimal. Second, we did not stratify the population according to the presence of high-risk co-morbidities. Yet, recent studies suggested that VE may be lower in people with underlying conditions compared to the general [28–31]. This limitation may have led to an overestimation of the number of events prevented by vaccination, as we may have attributed a potentially higher effectiveness to individuals with co-morbidities. However, a previous study looking at the prioritization of vaccination strategies in France, showed that accounting for age and comorbidities only made a small difference compared to accounting for age only, especially when the number of available doses was high [32]. Third, the vaccine effectiveness values used as input have several limiting elements. Our study only considered VE of mRNA vaccines since they were preferentially recommended in France, even though 12% of vaccine doses administered to people aged 50 years or above in France were not mRNA vaccines (as of March 6, 2022). Previous VE studies had shown that the effectiveness of adenoviral vector vaccines was slightly lower than that of mRNA vaccines. However, a booster dose of an mRNA vaccine may enhance vaccine protection in people who have initially received an adenoviral vector vaccine [33, 34]. Then, we made a strong assumption of a linear decrease in VE over time, with decreasing effectiveness after 13 weeks for a primary dose and 21 weeks for subsequent doses, covering a period of over 1 year after the dose, while the literature has only studied the decrease in VE up to nine months after vaccination [24]. Despite being an extrapolation of the decline in immunity, our assumption was based on data available in the literature (Additional file 1: Table S1) and was more realistic than using a constant VE. Fourth, for Alpha and Delta variants, we used VE estimated from an analysis conducted on the French population, using the same age groups as in our study, which limited biases [12]. However, estimates of VE against the Omicron BA.1 variant were not robust due to the wide confidence intervals observed around the point estimates. To mitigate this issue, we chose to incorporate more robust estimates from other countries for VE against severe forms related to the Omicron BA.1 variant [16, 21]. However, the age profiles of the populations analysed in these considered studies differed from that of our own study; this may have led to an over- or underestimation of the number of events prevented by vaccination.

Finally, our study has limitations that stem from the method itself, as it only accounted for the individual-level direct effects of vaccination on severe outcomes in vaccinated people. It ignored its indirect effects such as its impact on transmission and herd immunity (indirect protection of unvaccinated individuals due to reduced spread of the virus in a highly immune population). Quantifying the indirect effects would require a dynamic transmission model [9]. In addition, vaccination, by reducing the number of severe outcomes, also alleviated the burden placed on the healthcare system, and therefore, probably reduced the fatality rate of COVID-19, and possibly other pathologies treated in hospitals. Therefore, our study likely underestimated the overall impact of vaccination (direct and indirect) and the true number of averted events. Since our approach was not based on a dynamic transmission model, another methodological limitation is that the counterfactual scenario without vaccination could not account for the dynamics of natural immunity in the population, changing behaviours in the population and the likely additional control measures that would have been implemented to control the epidemic in the absence of vaccination.

## Conclusions

In conclusion, this study demonstrated the strong impact of vaccination on COVID-19 morbidity and mortality in France, in particular during the circulation of the Delta and Omicron BA.1 variants. These findings highlight the fundamental role that vaccination played to protect the population from severe outcomes associated with COVID-19, to reduce the strain on the healthcare system as well as the economic and societal costs associated with non-pharmaceutical measures. The estimates that we provided could serve as a basis for studies assessing the cost-effectiveness of COVID-19 vaccination, which can be useful for policymakers.

### Supplementary Information

**Additional file 1: Text S1.** Estimating the number of directly averted events. **Text S2.** Reconstructing time series of hospitalizations, ICU admissions and deaths by variant. **Table S1.** Estimations of vaccine effectiveness by Santé publique France (methodology described in Tamandjou et al., Vaccine 2023, DOI: 10.1016/j.vaccine.2023.02.062). **Table S2.** Estimations of vaccine effectiveness against hospitalizations in England, among people 65 years and above (Stowe et al., Nature Communications 2022, DOI: 10.1038/s41467-022-33378-7). **Table S3.** Estimations of vaccine effectiveness against deaths in Canada, among people aged 18 years and above (Buchan et al., JAMA Open Network 2022, DOI: 10.1001/jamanetworkopen.2022.32760). **Table S4.** Estimated number of averted hospitalizations by age, dose and variant in the French population aged 50 years and above, from week 53-2020 to week 9-2022. **Table S5.** Estimated number of averted ICU admissions by age, dose and variant in the French population aged 50 years and above, from week 53-2020 to week 9-2022. **Table S6.** Estimated number of averted deaths by age, dose and variant in the French population aged 50 years and above, from week 53-2020 to week 9-2022. **Figure S1.** Flowchart of the French vaccinated population aged 50 years and above included in our study, from week 53-2020 to week 9-2022 (VAC-SI database, Santé publique France). **Figure S2.** Vaccine coverage (A) and proportion of vaccinated people according to the week in which they received their last dose (the last dose at the time of observation), for 4 weeks of observation w (week 6-2021 (B), week 20-2021 (C), week 40-2021 (D) and week 9-2022 (E)). **Figure S3.** Numbers of hospitalizations (A), ICU admissions (B) and deaths (C) observed and expected without vaccination, in the French population aged 50 years and above, from week 53-2020 to week 9-2022.

## Data Availability

The aggregated datasets used and/or analysed during the current study are available from the corresponding author on reasonable request.

## Abbreviations

COVID-19: Coronavirus disease 2019
ICU: Intensive care unit
mRNA: Messenger ribonucleic acid
SARS-CoV-2: Severe acute respiratory syndrome coronavirus 2
VC: Vaccine coverage
VE: Vaccine effectiveness
