# Supplementary material for: Impact of vaccination against severe COVID-19 in the French population aged 50 years and above: a retrospective population-based study
Source: BMC Med. 2023 Nov 8;21:426. doi: 10.1186/s12916-023-03119-8 (PMC10633992; doi:10.1186/s12916-023-03119-8)
Supplement: Supplementary file 1 — Additional file 1: Text S1. Estimating the number of directly averted events. Text S2. Reconstructing time series of hospitalizations, ICU admissions and deaths by variant. Table S1. Estimations of vaccine effectiveness by Santé publique France (methodology described in Tamandjou et al., Vaccine 2023, DOI: 10.1016/j.vaccine.2023.02.062). Table S2. Estimations of vaccine effectiveness against hospitalizations in England, among people 65 years and above (Stowe et al., Nature Communications 2022, DOI: 10.1038/s41467-022-33378-7). Table S3. Estimations of vaccine effectiveness against deaths in Canada, among people aged 18 years and above (Buchan et al., JAMA Open Network 2022, DOI: 10.1001/jamanetworkopen.2022.32760). Table S4. Estimated number of averted hospitalizations by age, dose and variant in the French population aged 50 years and above, from week 53-2020 to week 9-2022. Table S5. Estimated number of averted ICU admissions by age, dose and variant in the French population aged 50 years and above, from week 53-2020 to week 9-2022. Table S6. Estimated number of averted deaths by age, dose and variant in the French population aged 50 years and above, from week 53-2020 to week 9-2022. Figure S1. Flowchart of the French vaccinated population aged 50 years and above included in our study, from week 53-2020 to week 9-2022 (VAC-SI database, Santé publique France). Figure S2. Vaccine coverage (A) and proportion of vaccinated people according to the week in which they received their last dose (the last dose at the time of observation), for 4 weeks of observation w (week 6-2021 (B), week 20-2021 (C), week 40-2021 (D) and week 9-2022 (E)). Figure S3. Numbers of hospitalizations (A), ICU admissions (B) and deaths (C) observed and expected without vaccination, in the French population aged 50 years and above, from week 53-2020 to week 9-2022. [file 12916_2023_3119_MOESM1_ESM.docx]

# Supplementary material

**Impact of vaccination against severe COVID-19 in the French population aged 50 years and above: a retrospective population-based study**

Laetitia Tan-Lhernould, Cynthia Tamandjou, Guilhem Deschamps, Johnny Platon, Cécile Sommen, Fanny Chereau, Isabelle Parent du Châtelet, Simon Cauchemez, Sophie Vaux*, Juliette Paireau*§

§ corresponding author. Email: juliette.paireau@santepubliquefrance.fr

* these authors contributed equally to this work.

**This PDF file includes:**

Texts S1 to S2

Tables S1 to S6

Figures S1 to S3

## Text S1: Estimating the number of directly averted events

The number of directly averted events $N_{averted}$ is the expected number of events without vaccination $N_{expected}$ multiplied by the proportion of the population that is directly protected by vaccination $P_{protected}$:

$N_{averted}= N_{expected} \times P_{protected}$ (1)

In addition, the expected number of events without vaccination $N_{expected}$ is the sum of the number of averted events $N_{averted}$ and the number of observed events $N_{observed}$:

$N_{expected}= N_{averted}+ N_{observed}$ (2)

Combining equations (1) and (2) gives:

$N_{averted}= N_{observed} \times\frac{P_{protected}}{1 - (P_{protected})}$ (3)

The equation is applied for each week w of the study period. For simplicity, we do not write the subscript w in all equations.

- In the simple case of a single-dose vaccine and a constant vaccine effectiveness (VE) over time, the proportion of the population protected by vaccination is the product of vaccine coverage ($VC$) and $VE$:

$P_{protected}=VC\times VE$ (4)

hence:

$N_{averted}= N_{observed} \times\frac{VC\times VE}{1 - (VC \times VE)}$ (5)

- In the case of a K-dose vaccine, the proportion of the population that is protected by vaccination is the sum over all doses of the proportion of the population protected by each dose:

$P_{protected}=\sum_{k=1}^{K} P_{protected, vaccinated with dose k}=\sum_{k=1}^{K} {VC}_{k}{\times VE}_{k}$ (6)

where ${VC}_{k}$represents the vaccine coverage of *exactly* $k$ doses (not *at least* $k$ doses), hence:

$N_{averted}= N_{observed} \times\frac{\sum_{k=1}^{K} {VC}_{k}{\times VE}_{k}}{1 - \sum_{k=1}^{K} {VC}_{k}{\times VE}_{k}}$ (7)

- In the case of a single-dose vaccine with varying VE over time, the proportion of the population protected by vaccination is the sum over all delays of the proportion of the population protected by vaccination since a given delay $:$

$P_{protected}=\sum_{\Delta=0}^{w-1} P_{protected, vaccinated since delay \Delta}=\sum_{\Delta=0}^{w-1} {VP}_{\Delta}{\times VE}_{\Delta}$ (8)

where ${VE}$ is the vaccine effectiveness weeks after vaccination, and ${VP}$ represents the proportion of people who received their dose exactly weeks ago ($\sum_{=0}^{w-1} {VP}={VC}_{w}).$ Hence:

$N_{averted}= N_{observed} \times\frac{\sum_{\Delta=0}^{w-1} {VC}_{\Delta}{\times VE}_{\Delta}}{1 - \sum_{\Delta=0}^{w-1} {VC}_{\Delta}{\times VE}_{\Delta}}$ (9)

Combining equations (7) and (9) (i.e. the case of a K-dose vaccine with varying VE over time) gives equation (3) presented in the main text and used in our study.

## Text S2: Reconstructing time series of hospitalizations, ICU admissions and deaths by variant

In order to reconstruct the time series of hospitalizations, ICU admissions and deaths by variant, we used SI-DEP database, which is the national surveillance system with records of all SARS-CoV-2 RT-PCR and antigen test results performed in all private and public French laboratories. Test results are reported by date of nasopharyngeal swab and include patient information such as age. Among positive RT-PCR or antigen test results, variants are identified based on the results of RT-PCR screening kits. The screening kits give information on the presence or absence of specific mutations: the presence of the L452R mutation was indicative of the Delta variant, while the presence of the deletion 69/70 or the mutations K417N, S371L-S373P or Q493R were indicative of the Omicron BA.1 variant. The SI-DEP database was linked to the SI-VIC database using a unique personal identifying number (a pseudonym – a character string constructed through concatenation and encryption of individuals’s surname, first name, sex and date of birth) in order to associate a variant to each hospitalized patient. This database was not exhaustive (not all patients could be matched) but it allowed us to estimate the weekly proportions of each variant in hospitalizations, ICU admissions and deaths, based on hospitalized patients for which screening results for the aforementioned mutations were known. We estimated the weekly proportions of Alpha, Delta and Omicron BA.1 within each age group, by dividing the study period into four periods based on the trends observed in the raw data (as shown in the figure below):

1. From December 28, 2020 to April 30, 2021, the weekly proportion of Alpha was set to 1 and the two other variants were set to 0. It should be noted that the historical strain was still circulating during the first months of the study period, but vaccine coverage was still low at that time (Additional file 1: Fig. S2); therefore, this approximation (setting Alpha to 1) should only have a limited impact on our estimates.
2. From May 1, 2021 to August 15, 2021, the weekly proportion of Delta was estimated by fitting a logistic regression model to the raw proportion of the Delta variant by week, in order to extract a smoothed trend from the raw (noisy) data. The proportion of Alpha was estimated as 1-Delta and the proportion of Omicron BA.1 was set to 0.
3. From August 16, 2021 to October 31, 2021, the weekly proportion of Delta was set to 1, while the two other variants were set to 0.
4. From November 1, 2021 to March 6, 2022, the weekly proportion of Omicron BA.1 was estimated by fitting a logistic regression model to the raw proportion of the Omicron BA.1 variant by week. The proportion of Delta was estimated as 1-Omicron and the proportion of Alpha was set to 0.

This procedure was applied separately for each age group and for each type of event (hospitalizations, ICU admissions and deaths).


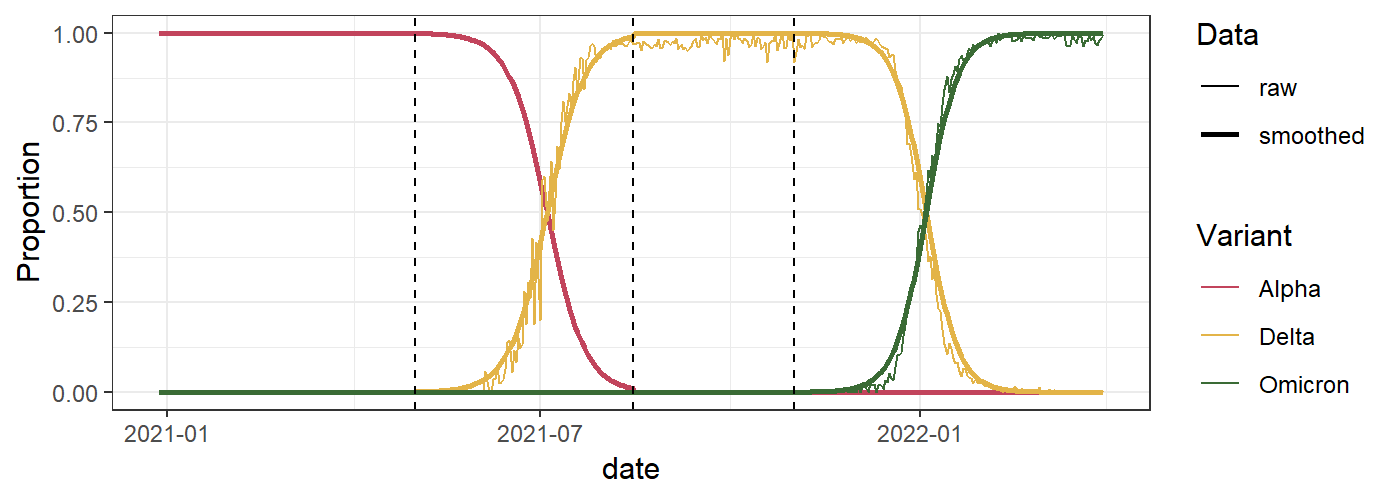


**Figure:** Raw and smoothed proportions of Alpha, Delta and Omicron BA.1 variants among hospitalizations in ≥ 50 years old population. The dashed lines delimit the four time periods.

Finally, the total number of observed hospitalizations, ICU admissions and deaths (obtained from the full SI-VIC database) was multiplied by the proportions of each variant in order to obtain time-series of hospitalizations, ICU admissions and deaths stratified by variant.

## Supplementary tables

**Table S1:** Estimations of vaccine effectiveness by Santé publique France (methodology described in Tamandjou *et al.*, Vaccine 2023, DOI: [10.1016/j.vaccine.2023.02.062](https://doi.org/10.1016/j.vaccine.2023.02.062))

| **Age group (years)** | **Variant of concern** | **Dose** | **Time since last dose (days)** | **Vaccine effectiveness against hospitalizations** | | | **Vaccine effectiveness against ICU admissions and deaths** | | |
| --- | --- | --- | --- | --- | --- | --- | --- | --- | --- |
|  |  |  |  | **Estimation** | **95%CI lower bound** | **95%CI upper bound** | **Estimation** | **95%CI lower bound** | **95%CI upper bound** |
| 50-79 | Alpha | One dose | 0-14 | **22.9%** | 12.9% | 37.4% | **25.4%** | 11.2% | 48.1% |
|  |  |  | 15-28 | **76.7%** | 63.9% | 85.9% | **79.8%** | 57.5% | 92.0% |
|  |  |  | >28 | **90.0%** | 48.0% | 98.9% | **79.9%** | 43.6% | 95.3% |
|  |  | Two doses | 0-7 | **87.3%** | 58.6% | 97.1% | **91.7%** | 15.3% | 99.9% |
|  |  |  | 8-30 | **97.1%** | 25.6% | 100.0% | **96.6%** | 20.1% | 100.0% |
|  |  |  | 31-60 | **94.7%** | 65.9% | 99.4% | **89.3%** | 56.9% | 98.1% |
|  |  |  | 61-90 | **95.1%** | 28.2% | 99.9% | **95.7%** | 2.4% | 100.0% |
|  |  |  | 91-120 | **100.0%** | 0.0% | NA | **100.0%** | 0.0% | NA |
|  |  |  | >120 | **100.0%** | 0.0% | NA | **100.0%** | 0.0% | NA |
|  | Delta | One dose | 0-14 | **44.8%** | 30.2% | 60.4% | **44.8%** | 30.2% | 60.4% |
|  |  |  | 15-28 | **82.3%** | 61.4% | 93.2% | **82.3%** | 61.4% | 93.2% |
|  |  |  | >28 | **96.7%** | 91.0% | 98.8% | **96.7%** | 91.0% | 98.8% |
|  |  | Two doses | 0-7 | **93.7%** | 44.2% | 99.6% | **93.7%** | 44.2% | 99.6% |
|  |  |  | 8-30 | **95.4%** | 85.8% | 98.6% | **95.4%** | 85.8% | 98.6% |
|  |  |  | 31-60- | **97.4%** | 68.3% | 99.9% | **97.4%** | 68.3% | 99.9% |
|  |  |  | 61-90 | **96.8%** | 79.3% | 99.6% | **96.8%** | 79.3% | 99.6% |
|  |  |  | 91-120 | **96.2%** | 83.5% | 99.2% | **96.2%** | 83.5% | 99.2% |
|  |  |  | >120 | **96.2%** | 90.5% | 98.5% | **96.2%** | 90.5% | 98.5% |
|  |  | First booster | 0-7 | **96.9%** | 57.6% | 99.9% | **96.9%** | 57.6% | 99.9% |
|  |  |  | 8-60 | **99.7%** | 58.9% | 100.0% | **99.7%** | 58.9% | 100.0% |
|  |  |  | 61-120 | **98.7%** | 75.4% | 100.0% | **98.7%** | 75.4% | 100.0% |
|  | Omicron | One dose | 0-14 |  |  |  | **43.3%** | 6.4% | 89.6% |
|  |  |  | 15-28 |  |  |  | **87.4%** | 0.7% | 100.0% |
| 80+ | Alpha | One dose | 0-14 | **-6.7%** | NA | NA | **-27.3%** | NA | NA |
|  |  |  | 15-28 | **50.8%** | 32.1% | 69.2% | **35.4%** | 13.4% | 66.1% |
|  |  |  | >28 | **73.3%** | 31.9% | 94.2% | **68.3%** | 19.9% | 94.9% |
|  |  | Two doses | 0-7 | **82.2%** | 47.6% | 95.9% | **80.6%** | 27.4% | 97.8% |
|  |  |  | 8-30 | **95.0%** | 40.8% | 99.8% | **90.6%** | 25.7% | 99.6% |
|  |  |  | 31-60 | **91.3%** | 60.0% | 98.7% | **87.7%** | 48.8% | 98.2% |
|  |  |  | 61-90 | **92.4%** | 32.8% | 99.7% | **96.4%** | 0.0% | 100.0% |
|  |  |  | 91-120 | **88.0%** | 0.4% | 100.0% | **67.7%** | 0.0% | 100.0% |
|  |  |  | >120 | **NA** | NA | NA | **NA** | NA | NA |
|  | Delta | One dose | 0-14 | **26.1%** | 3.4% | 78.0% | **15.6%** | 0.2% | 94.5% |
|  |  |  | 15-28 | **59.5%** | 19.2% | 90.1% | **76.1%** | 5.0% | 99.5% |
|  |  |  | >28 | **90.0%** | 62.6% | 98.0% | **86.4%** | 52.2% | 97.4% |
|  |  | Two doses | 0-7 | **83.0%** | 18.9% | 99.0% | **82.7%** | 8.1% | 99.6% |
|  |  |  | 8-30 | **96.2%** | 12.1% | 100.0% | **97.8%** | 0.0% | 100.0% |
|  |  |  | 31-60 | **91.8%** | 51.7% | 99.2% | **92.4%** | 24.3% | 99.8% |
|  |  |  | 61-90 | **89.3%** | 60.9% | 97.8% | **89.6%** | 39.9% | 99.1% |
|  |  |  | 91-120 | **86.1%** | 67.2% | 94.9% | **92.0%** | 28.9% | 99.7% |
|  |  |  | >120 | **81.6%** | 70.8% | 89.0% | **84.0%** | 65.1% | 93.6% |
|  |  | First booster | 0-7 | **83.5%** | 58.7% | 94.7% | **91.6%** | 12.6% | 99.9% |
|  |  |  | 8-60 | **98.3%** | 80.0% | 99.9% | **99.0%** | 20.2% | 100.0% |
|  |  |  | 61-120 | **99.2%** | 6.8% | 100.0% | **99.2%** | 0.0% | 100.0% |
|  | Omicron | One dose | 0-14 |  |  |  | **52.3%** | 6.2% | 94.8% |
|  |  |  | 15-28 |  |  |  | **72.2%** | 3.0% | 99.6% |
|  |  |  | >28 |  |  |  | **83.6%** | 8.4% | 99.7% |

**Table S2:** Estimations of vaccine effectiveness against hospitalizations in England, among people 65 years and above (Stowe *et al.*, Nature Communications 2022, DOI: [10.1038/s41467-022-33378-7](https://doi.org/10.1038/s41467-022-33378-7))

| **Variant of concern** | **Dose** | **Time since dose (days)** | **Vaccine effectiveness against hospitalizations** | | |
| --- | --- | --- | --- | --- | --- |
|  |  |  | **Estimation** | **95%CI lower bound** | **95%CI upper bound** |
| Omicron | One dose | 0-27 | **57.4%** | -0.7% | 82.2% |
|  |  | >27 | **52.3%** | 35.8% | 64.5% |
|  | Two doses | 14-174 | **80.5%** | 72.2% | 86.3% |
|  |  | >174 | **58.4%** | 51.0% | 64.7% |
|  | First booster | 0-6 | **78.5%** | 66.8% | 86.1% |
|  |  | 7-13 | **82.2%** | 73.0% | 88.3% |
|  |  | 14-34 | **91.3%** | 89.1% | 93.0% |
|  |  | 35-69 | **88.9%** | 87.1% | 90.6% |
|  |  | 70-104 | **87.6%** | 85.6% | 89.3% |
|  |  | >104 | **84.1%** | 81.2% | 86.5% |

**Table S3:** Estimations of vaccine effectiveness against deaths in Canada, among people aged 18 years and above (Buchan *et al.*, JAMA Network Open 2022, DOI: [10.1001/jamanetworkopen.2022.32760](https://doi.org/10.1001/jamanetworkopen.2022.32760))

| **Variant of concern** | **Dose** | **Time since dose (days)** | **Vaccine effectiveness against deaths** | | |
| --- | --- | --- | --- | --- | --- |
|  |  |  | **Estimation** | **95%CI lower bound** | **95%CI upper bound** |
| Omicron | Two doses | 7-59 | **89.0%** | 64.0% | 97.0% |
|  |  | 60-119 | **85.0%** | 69.0% | 92.0% |
|  |  | 120-179 | **92.0%** | 89.0% | 95.0% |
|  |  | 180-239 | **93.0%** | 89.0% | 95.0% |
|  |  | >239 | **93.0%** | 77.0% | 98.0% |
|  | First booster | 0-6 | **94.0%** | 90.0% | 96.0% |
|  |  | >6 | **98.0%** | 97.0% | 99.0% |

**Table S4:** Estimated number of averted hospitalizations by age, dose and variant in the French population aged 50 years and above, from week 53-2020 to week 9-2022. Results for the 95% confidence intervals are given in parenthesis.

| **Age group (years)** | **Variant of concern** | **Vaccine coverage (%) in week 9-2022** | | | **Number of hospitalizations** | | | | | **Hospitalization rate per 100,000** | | **% averted by vaccination** | |
| --- | --- | --- | --- | --- | --- | --- | --- | --- | --- | --- | --- | --- | --- |
|  |  | **One dose** | **Two doses** | **First booster** | **Observed** | **Averted in people vaccinated with one dose** | **Averted in people vaccinated with two doses** | **Averted in people vaccinated with the first booster** | **Total averted** | **Observed** | **Expected without vaccination** |  |  |
| **50-59** | **Alpha** | 93.8 | 90.5 | 77.4 | 25074 | 1792 (1011-2242) | 1485 (406-1636) | 0 (0-0) | 3277 (1417-3878) | 282 | 318.9 (298-325.6) | 11.6 (5.3-13.4) |  |
|  | **Delta** |  |  |  | 12496 | 6042 (3100-7403) | 34795 (16296-41545) | 8842 (2447-9878) | 49679 (21843-58826) | 140.6 | 699.3 (386.2-802.2) | 79.9 (63.6-82.5) |  |
|  | **Omicron** |  |  |  | 6187 | 458 (185-701) | 3716 (2781-4627) | 14396 (11708-17090) | 18570 (14674-22418) | 69.6 | 278.5 (234.6-321.7) | 75 (70.3-78.4) |  |
|  | **All** |  |  |  | 43757 | 8292 (4296-10346) | 39996 (19483-47808) | 23238 (14155-26968) | 71526 (37934-85122) | 492.2 | 1296.7 (918.8-1449.6) | 62 (46.4-66) |  |
| **60-69** | **Alpha** | 92.4 | 90.1 | 81.2 | 33228 | 3706 (1972-4747) | 2519 (628-2883) | 0 (0-0) | 6225 (2600-7630) | 412 | 489.1 (444.2-506.5) | 15.8 (7.3-18.7) |  |
|  | **Delta** |  |  |  | 15920 | 6016 (2982-7295) | 43103 (19779-51293) | 19697 (4980-21641) | 68816 (27741-80229) | 197.4 | 1050.5 (541.3-1192) | 81.2 (63.5-83.4) |  |
|  | **Omicron** |  |  |  | 10098 | 530 (210-808) | 4138 (3167-5064) | 27933 (23402-32428) | 32601 (26779-38300) | 125.2 | 529.4 (457.2-600) | 76.4 (72.6-79.1) |  |
|  | **All** |  |  |  | 59246 | 10252 (5164-12850) | 49760 (23574-59240) | 47630 (28382-54069) | 107642 (57120-126159) | 734.5 | 2069 (1442.7-2298.6) | 64.5 (49.1-68) |  |
| **70-79** | **Alpha** | 91.2 | 89.4 | 81.9 | 37076 | 4371 (2067-5805) | 8555 (1785-9961) | 0 (0-0) | 12926 (3852-15766) | 595.2 | 802.8 (657.1-848.4) | 25.9 (9.4-29.8) |  |
|  | **Delta** |  |  |  | 16248 | 4018 (1928-4800) | 39547 (18209-46683) | 30842 (7829-33688) | 74407 (27966-85171) | 260.9 | 1455.4 (709.8-1628.2) | 82.1 (63.3-84) |  |
|  | **Omicron** |  |  |  | 14717 | 529 (203-803) | 4460 (3478-5377) | 42540 (36416-48574) | 47529 (40097-54754) | 236.3 | 999.3 (880-1115.3) | 76.4 (73.2-78.8) |  |
|  | **All** |  |  |  | 68041 | 8918 (4198-11408) | 52562 (23472-62021) | 73382 (44245-82262) | 134862 (71915-155691) | 1092.4 | 3257.5 (2246.9-3591.9) | 66.5 (51.4-69.6) |  |
| **80+** | **Alpha** | 89.2 | 86.6 | 74.9 | 57352 | 2558 (970-11330) | 17417 (4572-24649) | 0 (0-0) | 19975 (5542-35979) | 1389.1 | 1872.8 (1523.3-2260.5) | 25.8 (8.8-38.5) |  |
|  | **Delta** |  |  |  | 20943 | 3222 (1013-4857) | 28495 (11138-40593) | 33147 (7074-40517) | 64864 (19225-85967) | 507.2 | 2078.2 (972.9-2589.3) | 75.6 (47.9-80.4) |  |
|  | **Omicron** |  |  |  | 30104 | 1123 (391-1746) | 10717 (8365-12876) | 69441 (59580-78976) | 81281 (68336-93598) | 729.1 | 2697.7 (2384.2-2996) | 73 (69.4-75.7) |  |
|  | **All** |  |  |  | 108399 | 6903 (2374-17933) | 56629 (24075-78118) | 102588 (66654-119493) | 166120 (93103-215544) | 2625.4 | 6648.8 (4880.3-7845.8) | 60.5 (46.2-66.5) |  |
| **Total**  **(50+)** | **Alpha** | 92.1 | 89.5 | 79.2 | 152730 | 12427 (6020-24124) | 29976 (7391-39129) | 0 (0-0) | 42403 (13411-63253) | 559.2 | 714.4 (608.3-790.7) | 21.7 (8.1-29.3) |  |
|  | **Delta** |  |  |  | 65607 | 19298 (9023-24355) | 145940 (65422-180114) | 92528 (22330-105724) | 257766 (96775-310193) | 240.2 | 1183.9 (594.5-1375.8) | 79.7 (59.6-82.5) |  |
|  | **Omicron** |  |  |  | 61106 | 2640 (989-4058) | 23031 (17791-27944) | 154310 (131106-177068) | 179981 (149886-209070) | 223.7 | 882.6 (772.5-989.1) | 74.7 (71-77.4) |  |
|  | **All** |  |  |  | 279443 | 34365 (16032-52537) | 198947 (90604-247187) | 246838 (153436-282792) | 480150 (260072-582516) | 1023.1 | 2780.9 (1975.2-3155.7) | 63.2 (48.2-67.6) |  |

**Table S5:** Estimated number of averted ICU admissions by age, dose and variant in the French population aged 50 years and above, from week 53-2020 to week 9-2022. Results for the 95% confidence intervals are given in parenthesis.

| **Age group (years)** | **Variant of concern** | **Vaccine coverage (%) in week 9-2022** | | | **Number of ICU admissions** | | | | | **Rate of ICU admissions per 100,000** | | **% averted by vaccination** |
| --- | --- | --- | --- | --- | --- | --- | --- | --- | --- | --- | --- | --- |
|  |  | **One dose** | **Two doses** | **First booster** | **Observed** | **Averted in people vaccinated with one dose** | **Averted in people vaccinated with two doses** | **Averted in people vaccinated with the first booster** | **Total averted** | **Observed** | **Expected without vaccination** |  |
| **50-59** | **Alpha** | 93.8 | 90.5 | 77.4 | 7677 | 554 (275-770) | 473 (49-534) | 0 (0-0) | 1027 (324-1304) | 86.3 | 97.9 (90-101) | 11.8 (4-14.5) |
|  | **Delta** |  |  |  | 4553 | 2259 (641-2864) | 12775 (2515-15627) | 3632 (366-4095) | 18666 (3522-22586) | 51.2 | 261.2 (90.8-305.3) | 80.4 (43.6-83.2) |
|  | **Omicron** |  |  |  | 1421 | 308 (0-427) | 1617 (1097-1920) | 6456 (4693-7539) | 8381 (5790-9886) | 16.0 | 110.3 (81.1-127.2) | 85.5 (80.3-87.4) |
|  | **All** |  |  |  | 13651 | 3121 (916-4061) | 14865 (3661-18081) | 10088 (5059-11634) | 28074 (9636-33776) | 153.5 | 469.3 (261.9-533.4) | 67.3 (41.4-71.2) |
| **60-69** | **Alpha** | 92.4 | 90.1 | 81.2 | 11543 | 1317 (619-1904) | 915 (90-1076) | 0 (0-0) | 2232 (709-2980) | 143.1 | 170.8 (151.9-180.1) | 16.2 (5.8-20.5) |
|  | **Delta** |  |  |  | 6297 | 2414 (679-3014) | 16989 (3489-20708) | 8524 (856-9443) | 27927 (5024-33165) | 78.1 | 424.3 (140.4-489.2) | 81.6 (44.4-84) |
|  | **Omicron** |  |  |  | 2335 | 362 (0-493) | 1800 (1297-2098) | 12469 (9670-14256) | 14631 (10967-16847) | 28.9 | 210.3 (164.9-237.8) | 86.2 (82.4-87.8) |
|  | **All** |  |  |  | 20175 | 4093 (1298-5411) | 19704 (4876-23882) | 20993 (10526-23699) | 44790 (16700-52992) | 250.1 | 805.4 (457.2-907.1) | 68.9 (45.3-72.4) |
| **70-79** | **Alpha** | 91.2 | 89.4 | 81.9 | 10887 | 1374 (541-2059) | 2685 (222-3214) | 0 (0-0) | 4059 (763-5273) | 174.8 | 240 (187-259.4) | 27.2 (6.5-32.6) |
|  | **Delta** |  |  |  | 5234 | 1292 (368-1584) | 12297 (2797-14835) | 10752 (1210-11855) | 24341 (4375-28274) | 84.0 | 474.8 (154.3-538) | 82.3 (45.5-84.4) |
|  | **Omicron** |  |  |  | 2562 | 274 (0-368) | 1468 (1113-1681) | 13865 (11344-15548) | 15607 (12457-17597) | 41.1 | 291.7 (241.1-323.6) | 85.9 (82.9-87.3) |
|  | **All** |  |  |  | 18683 | 2940 (909-4011) | 16450 (4132-19730) | 24617 (12554-27403) | 44007 (17595-51144) | 299.9 | 1006.5 (582.4-1121) | 70.2 (48.5-73.2) |
| **80+** | **Alpha** | 89.2 | 86.6 | 74.9 | 4285 | 159 (32-888) | 1336 (148-2054) | 0 (0-0) | 1495 (180-2942) | 103.8 | 140 (108.1-175) | 25.9 (4-40.7) |
|  | **Delta** |  |  |  | 1854 | 310 (43-505) | 3116 (266-4465) | 3021 (87-3656) | 6447 (396-8626) | 44.9 | 201 (54.5-253.8) | 77.7 (17.6-82.3) |
|  | **Omicron** |  |  |  | 1538 | 160 (2-229) | 975 (753-1119) | 6208 (5147-6939) | 7343 (5902-8287) | 37.3 | 215.1 (180.2-238) | 82.7 (79.3-84.3) |
|  | **All** |  |  |  | 7677 | 629 (77-1622) | 5427 (1167-7638) | 9229 (5234-10595) | 15285 (6478-19855) | 185.9 | 556.1 (342.8-666.8) | 66.6 (45.8-72.1) |
| **Total**  **(50+)** | **Alpha** | 92.1 | 89.5 | 79.2 | 34392 | 3404 (1467-5621) | 5409 (509-6878) | 0 (0-0) | 8813 (1976-12499) | 125.9 | 158.2 (133.1-171.7) | 20.4 (5.4-26.7) |
|  | **Delta** |  |  |  | 17938 | 6275 (1731-7967) | 45177 (9067-55635) | 25929 (2519-29049) | 77381 (13317-92651) | 65.7 | 349 (114.4-404.9) | 81.2 (42.6-83.8) |
|  | **Omicron** |  |  |  | 7856 | 1104 (2-1517) | 5860 (4260-6818) | 38998 (30854-44282) | 45962 (35116-52617) | 28.8 | 197 (157.3-221.4) | 85.4 (81.7-87) |
|  | **All** |  |  |  | 60186 | 10783 (3200-15105) | 56446 (13836-69331) | 64927 (33373-73331) | 132156 (50409-157767) | 220.3 | 704.2 (404.9-797.9) | 68.7 (45.6-72.4) |

**Table S6:** Estimated number of averted deaths by age, dose and variant in the French population aged 50 years and above, from week 53-2020 to week 9-2022. Results for the 95% confidence intervals are given in parenthesis.

| **Age group (years)** | **Variant of concern** | **Vaccine coverage (%) in week 9-2022** | | | **Number of deaths** | | | | | **Mortality rate per 100,000** | | **% averted by vaccination** |
| --- | --- | --- | --- | --- | --- | --- | --- | --- | --- | --- | --- | --- |
|  |  | **One dose** | **Two doses** | **First booster** | **Observed** | **Averted in people vaccinated with one dose** | **Averted in people vaccinated with two doses** | **Averted in people vaccinated with the first booster** | **Total averted** | **Observed** | **Expected without vaccination** |  |
| **50-59** | **Alpha** | 93.8 | 90.5 | 77.4 | 1545 | 102 (48-142) | 116 (10-129) | 0 (0-0) | 218 (58-271) | 17.4 | 19.8 (18-20.4) | 12.4 (3.6-14.9) |
|  | **Delta** |  |  |  | 901 | 366 (108-451) | 2144 (428-2578) | 920 (100-1003) | 3430 (636-4032) | 10.1 | 48.7 (17.3-55.5) | 79.2 (41.4-81.7) |
|  | **Omicron** |  |  |  | 393 | 71 (0-94) | 351 (249-410) | 1782 (1342-2046) | 2204 (1591-2550) | 4.4 | 29.2 (22.3-33.1) | 84.9 (80.2-86.6) |
|  | **All** |  |  |  | 2839 | 539 (156-687) | 2611 (687-3117) | 2702 (1442-3049) | 5852 (2285-6853) | 31.9 | 97.8 (57.6-109) | 67.3 (44.6-70.7) |
| **60-69** | **Alpha** | 92.4 | 90.1 | 81.2 | 4736 | 440 (203-631) | 435 (40-502) | 0 (0-0) | 875 (243-1133) | 58.7 | 69.6 (61.7-72.8) | 15.6 (4.9-19.3) |
|  | **Delta** |  |  |  | 2414 | 735 (215-896) | 5197 (1083-6245) | 4093 (445-4451) | 10025 (1743-11592) | 29.9 | 154.2 (51.5-173.6) | 80.6 (41.9-82.8) |
|  | **Omicron** |  |  |  | 1020 | 132 (0-177) | 644 (477-742) | 5161 (4105-5829) | 5937 (4582-6748) | 12.6 | 86.3 (69.5-96.3) | 85.3 (81.8-86.9) |
|  | **All** |  |  |  | 8170 | 1307 (418-1704) | 6276 (1600-7489) | 9254 (4550-10280) | 16837 (6568-19473) | 101.3 | 310 (182.7-342.7) | 67.3 (44.6-70.4) |
| **70-79** | **Alpha** | 91.2 | 89.4 | 81.9 | 9470 | 783 (288-1192) | 2201 (177-2585) | 0 (0-0) | 2984 (465-3777) | 152 | 199.9 (159.5-212.7) | 24 (4.7-28.5) |
|  | **Delta** |  |  |  | 3980 | 789 (242-953) | 7729 (1843-9223) | 8216 (1017-8917) | 16734 (3102-19093) | 63.9 | 332.6 (113.7-370.4) | 80.8 (43.8-82.8) |
|  | **Omicron** |  |  |  | 2143 | 203 (0-268) | 1064 (821-1209) | 11151 (9263-12411) | 12418 (10084-13888) | 34.4 | 233.8 (196.3-257.4) | 85.3 (82.5-86.6) |
|  | **All** |  |  |  | 15593 | 1775 (530-2413) | 10994 (2841-13017) | 19367 (10280-21328) | 32136 (13651-36758) | 250.3 | 766.3 (469.5-840.5) | 67.3 (46.7-70.2) |
| **80+** | **Alpha** | 89.2 | 86.6 | 74.9 | 31505 | 514 (97-3740) | 6547 (773-9603) | 0 (0-0) | 7061 (870-13343) | 763 | 934.1 (784.1-1086.2) | 18.3 (2.7-29.8) |
|  | **Delta** |  |  |  | 8879 | 1212 (188-1911) | 12144 (1096-16857) | 14905 (437-17371) | 28261 (1721-36139) | 215 | 899.5 (256.7-1090.3) | 76.1 (16.2-80.3) |
|  | **Omicron** |  |  |  | 7704 | 726 (17-1030) | 4499 (3516-5123) | 30004 (25164-33318) | 35229 (28697-39471) | 186.6 | 1039.8 (881.6-1142.6) | 82.1 (78.8-83.7) |
|  | **All** |  |  |  | 48088 | 2452 (302-6681) | 23190 (5385-31583) | 44909 (25601-50689) | 70551 (31288-88953) | 1 164.7 | 2873.4 (1922.5-3319.1) | 59.5 (39.4-64.9) |
| **Total**  **(50+)** | **Alpha** | 92.1 | 89.5 | 79.2 | 47256 | 1839 (636-5705) | 9299 (1000-12819) | 0 (0-0) | 11138 (1636-18524) | 173.0 | 213.8 (179-240.8) | 19.1 (3.3-28.2) |
|  | **Delta** |  |  |  | 16174 | 3102 (753-4211) | 27214 (4450-34903) | 28134 (1999-31742) | 58450 (7202-70856) | 59.2 | 273.2 (85.6-318.6) | 78.3 (30.8-81.4) |
|  | **Omicron** |  |  |  | 11260 | 1132 (17-1569) | 6558 (5063-7484) | 48098 (39874-53604) | 55788 (44954-62657) | 41.2 | 245.5 (205.8-270.6) | 83.2 (80-84.8) |
|  | **All** |  |  |  | 74690 | 6073 (1406-11485) | 43071 (10513-55206) | 76232 (41873-85346) | 125376 (53792-152037) | 273.4 | 732.5 (470.4-830.1) | 62.7 (41.9-67.1) |

## Supplementary figures

**
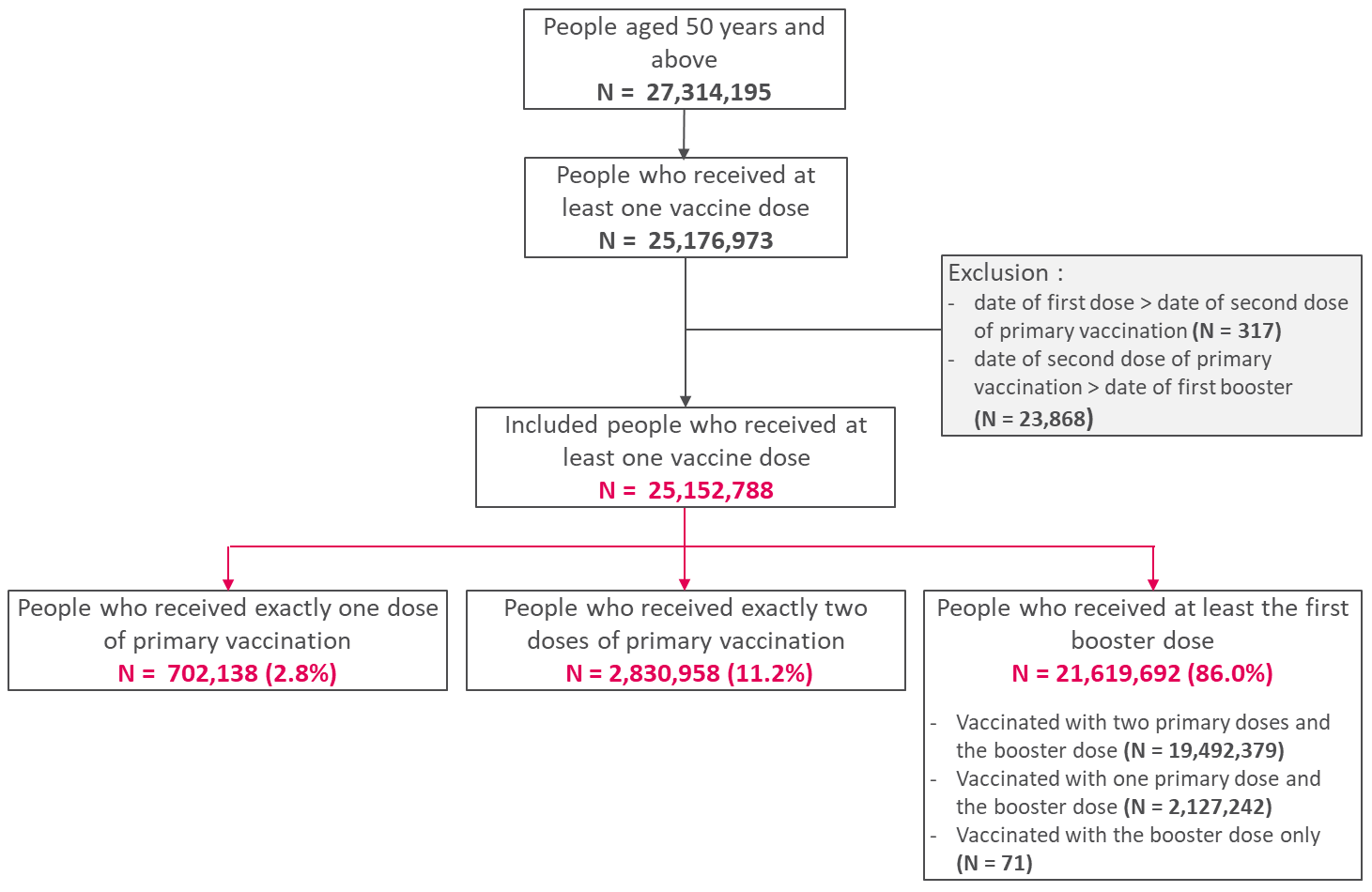
**

**Figure S1:** Flowchart of the French vaccinated population aged 50 years and above included in our study, from week 53-2020 to week 9-2022 (VAC-SI database, Santé publique France)


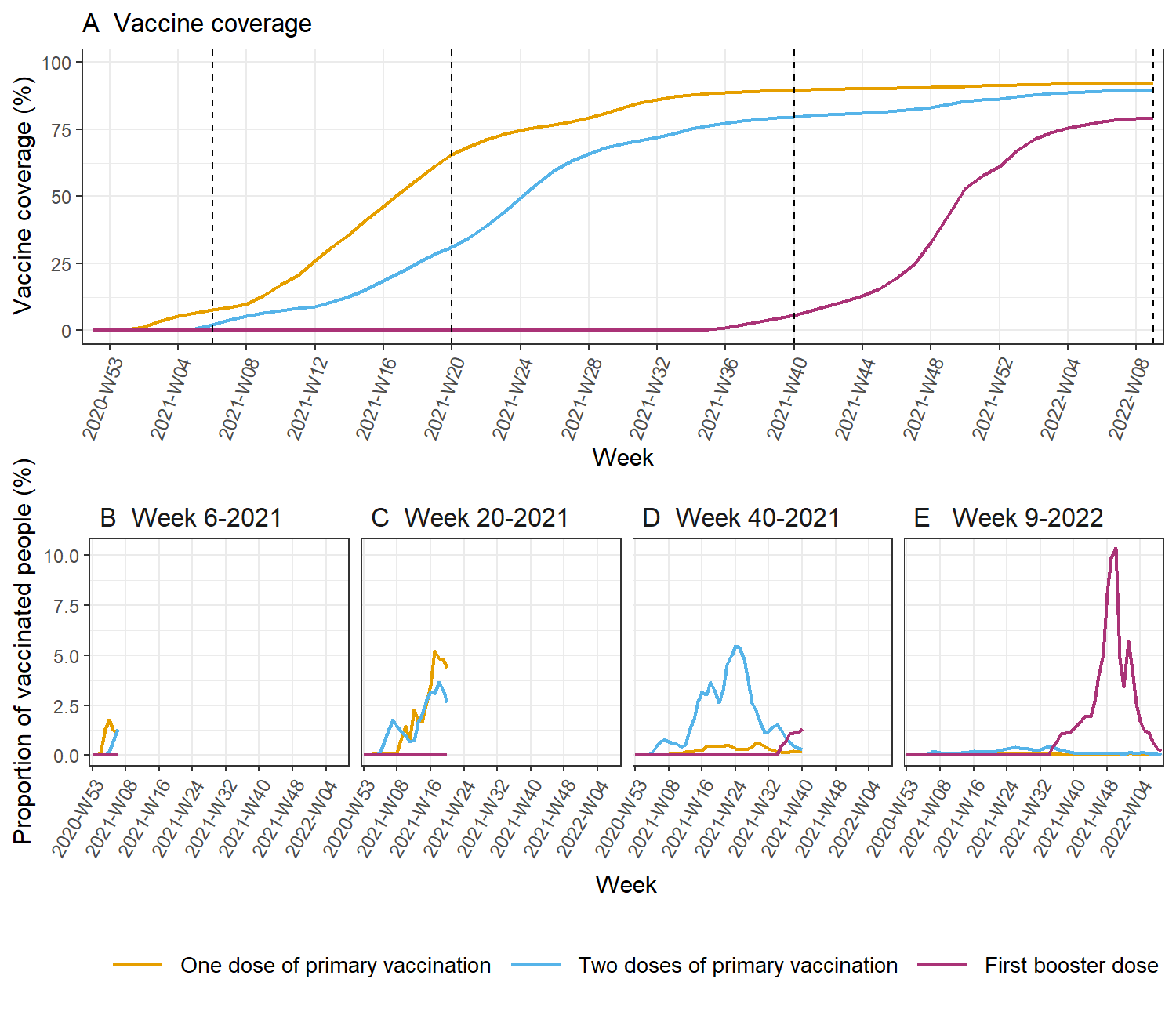


**Figure S2:** Vaccine coverage (A) and proportion of vaccinated people according to the week in which they received their last dose (the last dose at the time of observation), for 4 weeks of observation $w$ (week 6-2021 (B), week 20-2021 (C), week 40-2021 (D) and week 9-2022 (E)) (i.e. term ${VP}_{w,k,}$ in equation (3) of the main text). The vertical dotted lines in panel A show the observation weeks used in panels B to E. For example, in the week of observation 20-2021 (C), most people vaccinated with only one dose (orange line) were recently vaccinated people (vaccinated between week 8-2021 and week 20-2021, i.e. less than 12 weeks ago); there were very few people vaccinated with one dose before week 8-2021 (i.e. more than 12 weeks ago) because these individuals had already received their second dose (blue line) by week 20-2021. In week 40-2021 (D), most people had received a second dose (only a few people remained vaccinated with only one dose); the peak of vaccination for the second dose occurred in week 24-2021 (corresponding to a time since vaccination = 16 weeks). In week 9-2022 (E), most people had received the first booster dose (only a few people remained vaccinated with only one or two doses); the peak of vaccination for the booster occurred in week 50-2021 (corresponding to a time since vaccination = 11 weeks).

**
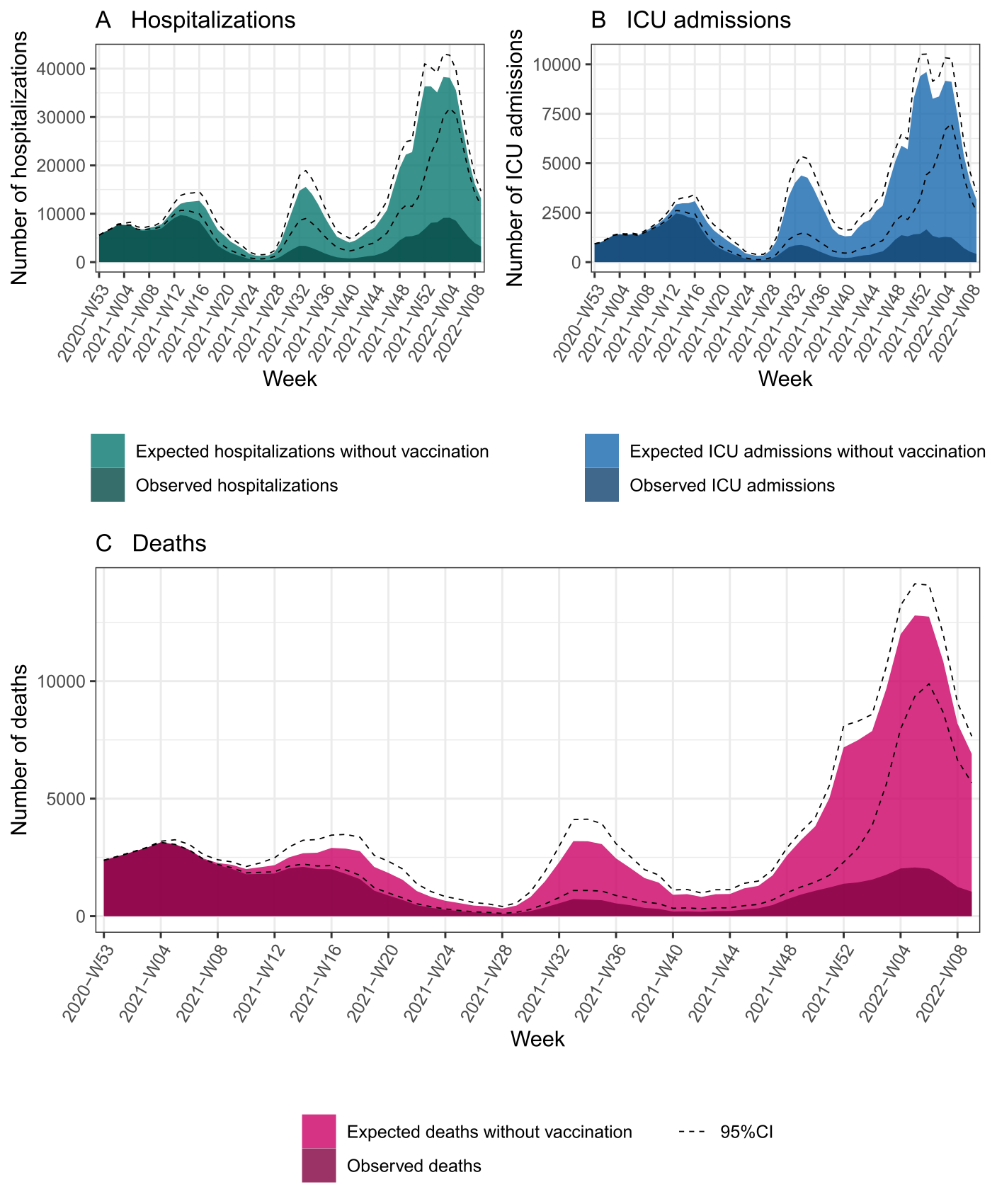
Figure S3:** Numbers of hospitalizations (A), ICU admissions (B) and deaths (C) observed and expected without vaccination, in the French population aged 50 years and above, from week 53-2020 to week 9-2022. Dashed lines show the 95% confidence intervals of the numbers of expected events.
